# Association between Toll-Like Receptor 4 and Occurrence of Type 2 Diabetes Mellitus Susceptible to Pulmonary Tuberculosis in Northeast China

**DOI:** 10.1155/2016/8160318

**Published:** 2016-03-31

**Authors:** Yuze Li, Dianzhong Li, Jinfeng Zhang, Shurui Liu, Haijun Chen, Kun Wu

**Affiliations:** ^1^Department of Nutrition and Food Hygiene, School of Public Health, Harbin Medical University, Harbin 150081, China; ^2^Department of the Fourth Internal Medicine, The Fourth Hospital of Heilongjiang Province, Harbin 150500, China; ^3^CT Department, Heilongjiang Province Hospital, Harbin 150036, China

## Abstract

The purpose of this study is to explore why type 2 diabetes mellitus (T2DM) patients are susceptible to pulmonary tuberculosis through detection of serum Toll-like receptor 4 (TLR_4_), an important immune-related receptor, especially in terms of content and TLR_4_ gene polymorphism. Patients with T2DM complicated by pulmonary tuberculosis (T2DMTB) were selected as the case group and T2DM patients without tuberculosis were selected as the control group. Forty patients in each group were randomly selected and their serum TLR_4_ levels were detected and compared. Determination of six sites of TLR_4_ gene polymorphism was carried out in 238 T2DMTB patients and 310 patients with T2DM, and results showed that the serum TLR_4_ content of the T2DMTB group was significantly lower than that of the T2DM group (*p* < 0.05). The six sites of TLR_4_ gene polymorphism did not show significant associations with T2DMTB risk. No statistically significant differences in genotype distributions were observed between T2DMTB patients and patients with T2DM when studied using the recessive and dominant genetic models. How two diseases with contradictory nutritional statuses can occur in the same person is difficult to explain from environmental factors perspective alone. Future research should study the causes of T2DMTB from the perspective of genetics.

## 1. Introduction

The prevalence of type 2 diabetes mellitus (T2DM) complicated by tuberculosis (TB) (T2DMTB) is rapidly increasing, resulting in detrimental effects on the economy and human health.

Clinical symptoms reveal that environmental factors play important roles in T2DM and TB. Because T2DMTB may be attributed to multiple causes, clinical experience indicates the traditional perspective that the two diseases are related in terms of nutrition. TB is associated with malnutrition, while T2DM is associated with excess nutrients. Thus, environmental factors, among which nutrition is one of the most important, alone, cannot explain the pathogenesis of two seemingly conflicting diseases occurring simultaneously in the same individual. In this case, genetic factors may be responsible for the development of T2DMTB [1–3], but we are not the first who are aware of host genetic factors that are important to determine the risk of type 2 diabetes mellitus complicated by tuberculosis. García-Elorriaga et al. [4] analyzed the association of inflammatory cytokine polymorphisms and T2DMTB in Mexico.

Toll-like receptor 4 (TLR_4_), a pattern recognition receptor, can recognize pathogen-associated molecular patterns (PAMPs) [5] and exacerbate and release inflammatory cytokines, such as TNF-*α*, IL-1*β*, IL-6, and IFN-*γ* [6].* Mycobacterium tuberculosis *presents liposaccharides (LPS), a type of PAMP. When TLR_4_ recognizes the LPS of* M. tuberculosis*, inflammatory cytokines are induced and released by the pathway of TLR_4_ [7–9]. This pathway is an important element in the innate immune system of the human body. TLR_4_, as an important mediator of inflammation, can recognize a variety of pathogens, including Gram-negative and Gram-positive bacteria [10]. Previous studies have shown that human TLR_4_ is a potentially important gene that may affect the onset of T2DM [11–13]. The receptor may be related to susceptibility to some infections that can induce diseases such as TBs [14–18]. To date, no study has yet reported the association between TLR_4_ gene polymorphisms and the risk of T2DMTB in the Chinese population.

We believe that TLR_4_ gene polymorphisms are a good starting point for studying T2DMTB. To be able to establish preventive measures for high-risk people as far as possible, in this study, we investigate the relationship between TLR_4_ gene polymorphisms and T2DMTB risk in Northeast China.

## 2. Material and Methods 

### 2.1. Study Subject and Sample Collection

We recruited 238 cases with T2DMTB as the case group and 310 cases with T2DM as the control group. T2DMTB patient information was obtained from the Fourth Medical Ward, Heilongjiang Province Tuberculosis Control Center, from September 2013 to June 2015. T2DM patient information was obtained from the Second Department of Outpatient Services, Heilongjiang Province Tuberculosis Control Center, from September 2013 to November 2014.

Subjects with a history of blood-transmitted diseases, such as AIDS, hepatitis B, hepatitis C, or other endocrine diseases, were excluded from this study. Blood samples were taken from the patients, and DNA was obtained and stored in 193 K. Both doctors and study subjects provided consent to participate in this work, and the Ethics Committee of Harbin Medical University approved of this research.

### 2.2. Immunohistochemical Method

To determine whether serum TLR_4_ levels were consistent with the gene expression level, 40 serum samples were collected from the T2DMTB group and T2DM group, respectively, according to the principle of random. Serum TLR_4_ contents were detected by using an immunohistochemical method following the manufacturer's instructions (Beijing Cheng Lin Biological Technology Co., Ltd.).

### 2.3. Tag SNP Selection and Genotyping

Tag SNPs of TLR_4_ gene were evaluated and selected by using the HapMap database (https://hapmap.ncbi.nlm.nih.gov/) with the following criterion: a minor allele frequency (MAF) > 0.05 in the Chinese Han population in the National Center of Biotechnology Information Database; linkage disequilibrium (LD) blocks were established by using Chinese LD maps and *r* ^2^ > 0.8. Six tag SNPs representing the genetic information of TLR_4_ were selected for genotyping: rs1927914 located in the promoter region; rs11536879, rs1927911, and rs1927907 located in the intron region; and rs11536889 and rs7873784 located in the 3′-UTR region.

According to the manufacturer's instructions, genomic DNA was extracted from peripheral blood using a Tiangen DNA Blood Mini kit (Tiangen Biotech Co., Ltd., Beijing, China). All SNPs were genotyped using fluorogenic 5′-nuclease assay (TaqMan SNP Genotyping Assay, Applied Biosystems, Foster City, CA, USA). For quality control, 20% of all of the samples were performed in delicate form randomly for each SNP. The concordance rate of these repeated samples was 100%.

### 2.4. Statistical Analysis

Numerical data are expressed as mean ± SD. Student's* t-*test was performed to analyze differences between the T2DMTB and T2DM groups.

Fisher's exact test was used to evaluate the Hardy-Weinberg equilibrium (HWE) in the subjects. The chi-square test or Fisher's exact test was used to identify statistical differences in the distributions of clinic pathological characteristics. Univariate and multivariate unconditional logistic regression were used to estimate crude and adjusted odds ratios (ORs) and 95% confidence intervals (CIs), all of which measured the associations between the risk factors of T2DMTB compared with T2DM. Two-sided *p* < 0.05 was considered as statistically significant. Statistical analysis was carried out by using SAS software (version 9.1.3, SAS Institute, Cary, NC).

## 3. Results

### 3.1. Demographic Characteristics

A total of 548 people participated in this study, including 238 cases of T2MDTB and 310 cases of T2MD. Among the patients, 338 were male and 210 were female. The mean ages of T2DMTB and T2DM patients were 51.97 ± 11.87 years and 58.73 ± 11.46 years, respectively. The demographic characteristics of the patients are shown in Table 1.

### 3.2. Serum TLR_4_ Levels of Patients with T2DMTB and Patients with T2DM

The serum TLR_4_ levels of subjects with T2DMTB were significantly lower than those of subjects suffering from T2DM (*p* < 0.0001). No differences in the basic characteristics of the participants, including gender (*p* = 0.260) and age (*p* = 0.631), were observed (Table 2).

### 3.3. Distributions of SNP Genotypes between Patients with T2DMTB and Patients with T2DM

Genotype distributions did not show statistically significant deviations from HWE for all SNPs in this study (Table 3).

### 3.4. Distribution of Allelic Genes of SNPs between Patients with T2DMTB and Patients with T2DM

The distributions of allelic genes of six SNPs of the TLR_4_ gene were not statistically significant between patients with T2DMTB and patients with T2DM. Details of these distributions are summarized in Table 4.

### 3.5. SNPs and T2DMTB Risk

The SNPs of TLR_4_ were not significantly associated with T2DMTB risk. No significant differences in genotype distributions between patients with T2DMTB and patients with T2DM were observed using the recessive and dominant genetic models (Table 5). However, potential trends may provide useful information for future studies. In rs7873784, compared with the GG genotype, the GC genotype presented lower risks of T2DMTB (OR_adjusted_ = 0.69, 95% CI: 0.42–1.14, *p* = 0.15). In rs11536879, AG genotype carriers showed decreased risk of T2DMTB compared with the GG genotype (OR_adjusted_ = 0.68, 95% CI: 0.43–1.08, *p* = 0.10).

## 4. Discussion 

A previous study showed that patients with T2DM demonstrated four to eight times increased risk of tuberculosis compared to patients without T2DM. For instance, TB in T2DM patients was 5 times more prevalent than in non-T2DM patients in some regions in the USA, 5.4 times more prevalent than in non-T2DM patients in Australia, and 6.8 times more prevalent than in non-T2DM patients in Mexico [4].

In the clinic, we discovered that T2DMTB patients present uneven changes in inflammatory cytokines such as TNF-*α* and IFN-*γ*. Clinical examination of simple TB yielded a positive IFN-*γ* test, but the results of nearly all patients with T2DMTB were negative. TLR_4_ is an important element of the endogenic immune system and it can induce and release inflammatory cytokines [5]. TLR_4_ is an important element of the innate immune system of the human body. Thus, we hypothesize that serum TLR_4_ exhibits obvious changes in T2DMTB patients. We examined TLR_4_ serum levels in patients with T2DMTB and patients T2DM to determine whether TLR_4_ is involved in the susceptibility of T2DM patients suffering from TB in Northeast China. While no study has yet proven that TLR_4_ polymorphisms are related to T2DMTB or T2DM, other studies [19–22] indicate that TLR_4_ polymorphisms present statistically significant differences between healthy people and patients with TB.

Our study results are different from those of Wu et al. [23], especially in terms of the frequencies of the GG genotype of SNP rs7873784 in TLR_4_ (OR = 2.136; 95% CI: 1.312–3.478) and the CC genotype of rs3764879 in TLR_8_ (OR = 1.982; 95% CI: 1.292–3.042). It was also significantly higher in the TB group than in the healthy group. Arji et al. [24] found TLR_4_ interactions influencing protection against TB in Moroccan patients. The present work and that of Jahantigh et al. [25] both demonstrated no significant relation between TLR_4_ and TLR_9_ polymorphisms and TB. Sánchez et al. [26] reported that they did not find any association between TLR_4_ polymorphic variants. These findings suggest that the gene polymorphisms were not involved in any risk factor for pulmonary TB in the Colombian population [26]. The same result was obtained by Xue et al. [27], who discovered that these polymorphisms were rare in the Southeastern Chinese population and not linked to susceptibility to TB. Newport et al. [28] also have studied that, but result was that no association between TLR_4_ Asp299Gly and TB was observed.

The result of our study showed that it was negative. The reasons behind this result are first the size of the sample which was not enough. The second one is the geographic regions and genetic factors, because we were focused on the people of Northeast China in our work just only.

The purpose of this study is to determine why patients with T2DM easily develop pulmonary TB. Thus, we did not detect TLR_4_ gene polymorphisms in healthy cases and we did not compare changes in TLR_4_ gene polymorphisms among healthy cases, patients with T2DM, and patients with T2DMTB. Changes in TLR_4_ gene polymorphisms may have already occurred in most patients with T2DM. In this case, a false negative result may appear if a comparison was carried out between T2DM patients and patients suffering from T2DMTB only. Such a phenomenon would also confirm some reports that T2DM is characterized by chronic inflammation.

Some studies have recently mentioned that the risk genotypes of rs1927914 are significantly linked with diabetic foot ulcers [29]; the research team of Singh [30] also found that the combined genotype risks of TLR_4_ SNPs rs10759931 (odds ratio [OR] 1.50, *p* = 0.05) and rs1927914 (OR 1.48, *p* = 0.05) were significantly linked to retinopathy in T2DM. These works support the idea that T2DM is a chronic inflammatory disease. It is possible that T2DM patients already had TLR_4_ gene polymorphism. Therefore, we considered these points as the third reason.

Two groups of patients were included in our study. For each group, we concerned about the changing of TLR_4_ gene polymorphism and no changing of TLR_4_ levels in the blood. The reason of that was hidden behind the changing in the TLR_4_ gene, because serum TLR_4_ is a sensitivity index but not a specific index. Chronic inflammation could directly lead to changes in TLR_4_ in the blood or other gene polymorphisms that could lead to TLR_4_ changes in the blood. Our study was limited by considering just TLR_4_ gene polymorphisms. Other inflammatory gene polymorphisms that promote development of T2DMTB may exist.

The results of this study reveal that TLR_4_ changes at the molecular level are insignificant because the changes at the gene level may be not significant. Our results also demonstrated no significant link between the six SNPs of TLR_4_ studied in this work and the susceptibility of patients with T2DMTB in Northeast China. Future studies may be performed to determine the causes of T2DMTB from the perspective of genetics. We aim to determine the marker gene polymorphism in T2DM and show how it can be complicated by TB.

## Figures and Tables

**Table 1:** Demographic characteristics of the subjects.

Variable	T2DMTB	T2DM	Total	*p* value
Age				
⩽50	104	68	172	<0.0001
50–60	73	108	181	
60–70	45	80	125	
*⩾*70	16	54	70	
Gender				
Male	167	171	338	0.0003
Female	71	139	210	
BMI				
⩽18.5	35	8	43	<0.0001
18.5–24	112	64	176	
*⩾*24	91	238	329	
Smoking				
Yes	132	86	218	<0.0001
No	106	224	330	
Drinking				
Yes	108	94	202	0.0003
No	130	216	346	
Glucose				
<5.0	12	13	25	0.0857
5.0–7.2	48	88	136	
>7.2	178	209	387	
Insulin use				
Use	112	196	308	<0.0001
No use	126	112	238	
Unclear	0	2	2	
Hypoglycemic drug use				
Use	45	224	269	<0.0001
No use	193	80	273	
Unclear	0	6	6	

**Table 2:** Serum TLR_4_ levels of patients with T2DMTB and T2DM.

Variable	T2DMTB	T2DM	*p* value
Gender (male/female)	40 (20/20)	40 (25/15)	0.260
Age (years)	51.17 ± 6.70	51.85 ± 6.20	0.631
TLR_4_ (ng/mL)	15.27 ± 2.52	19.40 ± 1.80	<0.0001

**Table 3:** Distributions of SNP genotypes and Hardy-Weinberg equilibrium.

	T2DMTB No. (%)	T2DM No. (%)	*p* value	Hardy-Weinberg	
				*χ* ^2^	*p* value
rs7873784					
GG	243 (86.17)	236 (83.69)	0.19	0.56	0.46
GC	35 (12.41)	45 (15.96)			
CC	4 (1.42)	1 (0.35)			
rs11536889					
GG	176 (61.97)	181 (64.41)	0.83	0.81	0.37
GC	93 (32.75)	86 (30.61)			
CC	15 (5.28)	14 (4.98)			
rs1927914					
TT	90 (31.91)	102 (36.17)	0.50	0.53	0.47
CT	145 (51.42)	140 (49.65)			
CC	47 (16.67)	40 (14.18)			
rs1927911					
TT	97 (34.28)	96 (34.16)	0.48	2.92	0.09
CT	139 (49.12)	148 (52.67)			
CC	47 (16.61)	37 (13.17)			
rs1927907					
GG	145 (51.24)	147 (52.31)	0.96	2.12	0.15
AG	123 (43.46)	119 (42.35)			
AA	15 (5.30)	15 (5.34)			
rs11536879					
AA	230 (82.44)	215 (77.62)	0.20	1.04	0.31
AG	41 (14.70)	56 (20.22)			
GG	8 (2.87)	6 (2.17)			

**Table 4:** Distributions of allelic genes of SNPs between patients with T2DMTB and patients with T2DM.

SNP	Allelic genes	T2DMTB		T2DM		*p* value
		No.	%	No.	%	
rs7873784	G allele	521	92.36	517	91.67	0.74
	C allele	43	7.64	47	8.33	

rs11536889	G allele	445	78.35	448	79.72	0.61
	C allele	123	21.65	114	20.28	

rs1927914	T allele	325	57.62	344	60.99	0.43
	C allele	229	42.38	220	39.01	

rs1927911	T allele	333	58.83	340	60.50	0.59
	C allele	233	41.17	222	39.50	

rs1927907	G allele	413	72.97	413	73.49	0.89
	A allele	153	27.03	149	26.51	

rs11536879	A allele	501	89.78	486	87.73	0.30
	G allele	57	10.22	68	12.27	

**Table 5:** Associations between the SNPs of TLR_4_ and DMTB risk.

Genotype	Crude OR (95% CI)	*p* value	Adjusted OR (95% CI)^*∗*^	*p* value
rs7873784				
GG	1.00		1.00	
GC	0.76 (0.47–1.22)	0.25	0.69 (0.42–1.14)	0.15
CC	3.89 (0.43–35.01)	0.23	3.16 (0.33–30.74)	0.32
CC/(GG + GC)	4.04 (0.45–36.41)	0.21	3.34 (0.34–32.38)	0.30
(GC + CC)/GG	0.82 (0.52–1.31)	0.41	0.75 (0.46–1.22)	0.25
rs11536889				
GG	1.00		1.00	
GC	1.12 (0.78–1.60)	0.54	1.04 (0.71–1.51)	0.85
CC	1.11 (0.52–2.36)	0.79	1.08 (0.49–2.39)	0.85
CC/(GG + GC)	1.07 (0.51–2.26)	0.86	1.07 (0.49–2.34)	0.87
(GC + CC)/GG	1.12 (0.79–1.57)	0.53	1.04 (0.73–1.49)	0.82
rs1927914				
TT	1.00		1.00	
CT	1.17 (0.81–1.69)	0.39	1.25 (0.85–1.84)	0.25
CC	1.33 (0.80–2.21)	0.27	1.35 (0.80–2.29)	0.27
CC/(TT + CT)	1.21 (0.77–1.91)	0.42	1.18 (0.73–1.91)	0.49
(CT + CC)/TT	1.21 (0.85–1.71)	0.29	1.28 (0.89–1.84)	0.19
rs1927911				
TT	1.00		1.00	
CT	0.93 (0.65–1.34)	0.70	0.93 (0.63–1.36)	0.71
CC	1.26 (0.75–2.10)	0.38	1.22 (0.72–2.09)	0.46
CC/(TT + CT)	1.31 (0.82–2.09)	0.25	1.28 (0.79–2.08)	0.36
(CT + CC)/TT	1.00 (0.70–1.41)	0.98	0.99 (0.69–1.43)	0.96
rs1927907				
GG	1.00		1.00	
AG	1.05 (0.75–1.47)	0.79	1.03 (0.72–1.48)	0.86
AA	1.01 (0.48–2.15)	0.97	0.95 (0.44–2.05)	0.89
AA/(GG + AG)	0.99 (0.48–2.07)	0.98	0.93 (0.44–1.99)	0.85
(AG + AA)/GG	1.04 (0.75–1.45)	0.80	1.02 (0.72–1.44)	0.90
rs11536879				
AA	1.00		1.00	
AG	0.68 (0.44–1.07)	0.09	0.68 (0.43–1.08)	0.10
GG	1.25 (0.43–3.65)	0.69	1.35 (0.45–4.04)	0.59
GG/(AA + AG)	1.33 (0.46–3.89)	0.60	1.44 (0.48–4.31)	0.51
(AG + GG)/AA	0.74 (0.49–1.12)	0.16	0.75 (0.48–1.15)	0.19

*Note*. ^*∗*^Age, gender, BMI, smoking, drinking, insulin use, and hypoglycemic drug use were adjusted.
